# Pokeweed Antiviral Protein Increases HIV-1 Particle Infectivity by Activating the Cellular Mitogen Activated Protein Kinase Pathway

**DOI:** 10.1371/journal.pone.0036369

**Published:** 2012-05-01

**Authors:** Sheila Mansouri, Meherzad Kutky, Katalin A. Hudak

**Affiliations:** Department of Biology, York University, Toronto, Ontario, Canada; Lady Davis Institute for Medical Research, Canada

## Abstract

Pokeweed antiviral protein (PAP) is a plant-derived *N*-glycosidase that exhibits antiviral activity against several viruses. The enzyme removes purine bases from the messenger RNAs of the retroviruses Human immunodeficiency virus-1 and Human T-cell leukemia virus-1. This depurination reduces viral protein synthesis by stalling elongating ribosomes at nucleotides with a missing base. Here, we transiently expressed PAP in cells with a proviral clone of HIV-1 to examine the effect of the protein on virus production and quality. PAP reduced virus production by approximately 450-fold, as measured by p24 ELISA of media containing virions, which correlated with a substantial decline in virus protein synthesis in cells. However, particles released from PAP-expressing cells were approximately 7-fold more infectious, as determined by single-cycle infection of 1G5 cells and productive infection of MT2 cells. This increase in infectivity was not likely due to changes in the processing of HIV-1 polyproteins, RNA packaging efficiency or maturation of virus. Rather, expression of PAP activated the ERK1/2 MAPK pathway to a limited extent, resulting in increased phosphorylation of viral p17 matrix protein. The increase in infectivity of HIV-1 particles produced from PAP-expressing cells was compensated by the reduction in virus number; that is, virus production decreased upon *de novo* infection of cells over time. However, our findings emphasize the importance of investigating the influence of heterologous protein expression upon host cells when assessing their potential for antiviral applications.

## Introduction

Pokeweed antiviral protein (PAP) is a type I ribosome inactivating protein isolated from the pokeweed plant, *Phytolacca americana* [1]. Like all ribosome inactivating proteins, PAP is an *N*-glycosidase that removes a specific adenine base from the conserved sarcin/ricin loop of the large ribosomal RNA [2]. Depurination of rRNA slows the elongation step of translation by inhibiting the binding of elongation factor 2 (EF-2) [3], [4]. Resulting cytotoxicity has been cited to explain the antiviral properties of some of these enzymes [5]; namely, host cell death would hinder virus proliferation. However, subsequent studies have shown that antiviral activity can be achieved in the absence of cell death. Specifically, treatment of HIV-1 infected T-lymphocytes with purified PAP reduced virus production without causing cytotoxicity [6]. Moreover, PAP depurinates Human immunodeficiency virus-1 (HIV-1) genomic RNA *in vitro* [7] and we have shown recently that depurination of Human T-cell leukemia virus-1 (HTLV-1) gag mRNA decreased its translational efficiency without reducing cellular translation rates [8]. These findings suggest that the substrate specificity of PAP is not limited to rRNA and that antiviral activity is due to depurination of viral RNAs and resulting inhibition of viral protein synthesis. Although PAP substantially reduces HIV-1 production from infected cells, its application as an anti-HIV-1 agent requires a clear understanding of its effects on other virus life-cycle events, such as infection of target cells, which reflects the quality of virus particles produced from cells exposed to PAP.

A number of HIV-1 particle characteristics are known to influence the infectivity of this virus. For example, the relative amounts of processed HIV-1 structural and enzymatic proteins, such as envelope glycoprotein (Env) [9], Gag [10] and reverse transcriptase (RT) [11] affect the degree of maturation, and thereby infectivity, of HIV-1 particles. The efficiency of genomic RNA packaging within virus particles is another factor known to significantly enhance virus infectivity by increasing the number of replication competent HIV-1 particles [12]. Several cellular proteins have also been reported within HIV-1 particles [13]. Although the precise roles of most of these in the HIV-1 life-cycle are not well understood, some have been found to affect virus infectivity. Specifically, HIV-1 packages serine-threonine kinases such as ERK2 MAPK [14], [15] and cellular protein kinase A (C-PKA) [16] upon budding from cells, and activation of these kinases by cytokines and mitogens inside virus-producing cells caused release of more infectious particles [15], [16]. ERK2 MAPK predominantly phosphorylates matrix (MA) protein [15], while C-PKA was shown to phosphorylate capsid (CA) protein *in vitro* [16]. MA phosphorylation increased the nuclear localization of preintegration complexes [17], whereas CA phosphorylation was required for complete reverse transcription, given that mutants of CA unable to undergo phosphorylation were impaired for reverse transcription [18]. Therefore, current data suggest that the incorporation of some activated cellular kinases in particles is required for optimal infectivity.

**Figure 1:**
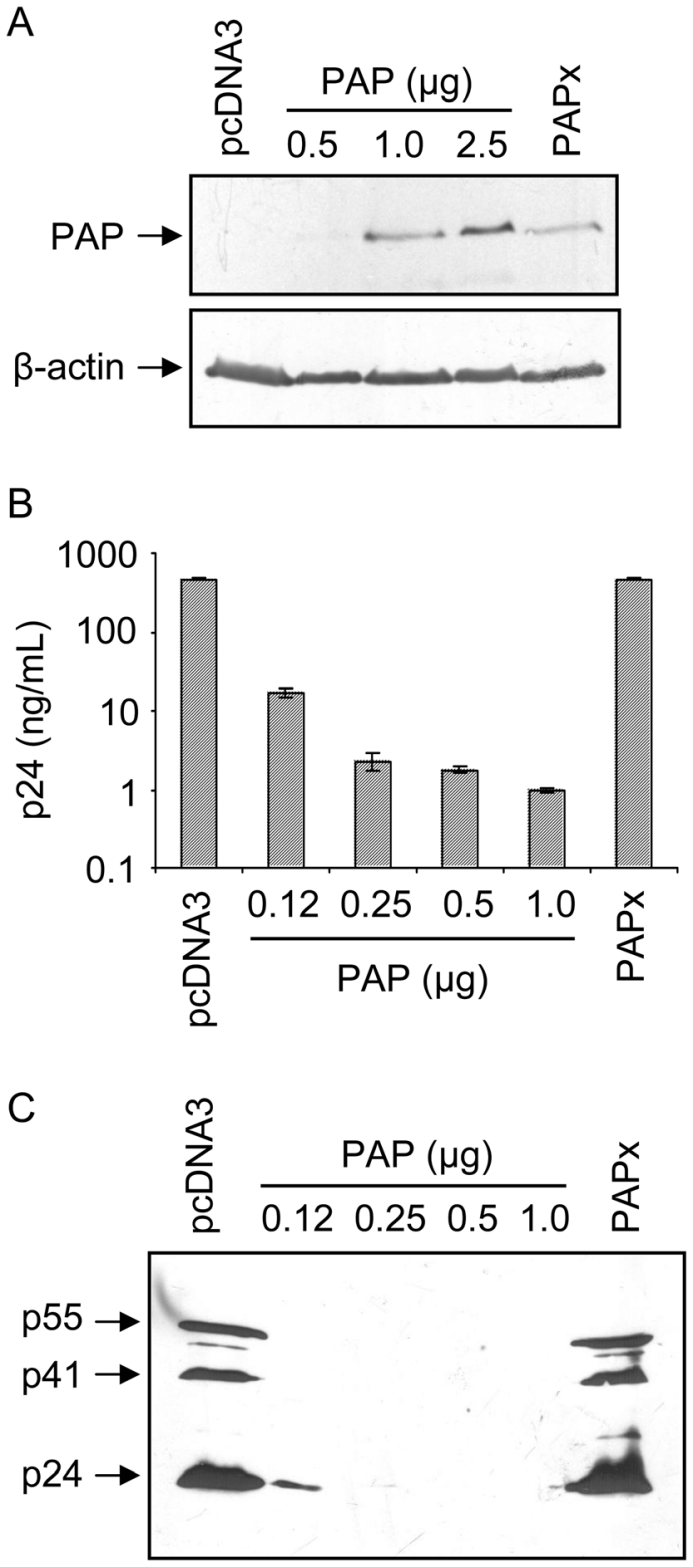
PAP reduces HIV-1 production from cells. (A) Immunoblot analysis of PAP expression in 293 T cells transfected with 3x-Flag-PAP (0.5, 1.0 or 2.5 µg), 3x-Flag-PAPx (0.5 µg) or pcDNA3 (2.5 µg) plasmids. Total cellular protein (100 µg) was resolved on a 12% SDS-PAGE, transferred to nitrocellulose and probed with Flag monoclonal antibody (1∶1,000) and β-actin monoclonal antibody (1∶5,000). (B) 293 T cells were transfected with pMenv(-) proviral clone (5 µg) and 3x-Flag-PAP (0.12, 0.25, 0.5 or 1.0 µg), 3x-Flag-PAPx (1.0 µg) or pcDNA3 (1.0 µg). Media of cells were collected 40 hours following transfection and virus production was estimated using a p24 CA ELISA. Values are plotted on log scale and are means ± S.E. from triplicate samples of three different experiments. (C) Equal volume of media (1 mL) was centrifuged and pelleted virus particles were separated through 12% SDS-PAGE followed by immunoblotting using a p24 CA-specific monoclonal antibody (1∶5,000). The blot is representative of three separate experiments.

In this study, we investigated the effects of PAP expression on HIV-1 production and infectivity. To determine whether PAP affects the quality of particles, we assessed the maturation of virion proteins and tested the efficiency of genomic RNA packaging within particles, and conclude that neither is altered by PAP. Surprisingly, PAP enhanced the infectivity of particles, which we attribute to activation of ERK2 kinase in virus particles, as shown by increased phosphorylation of MA protein during an *in vitro* kinase assay. Though we predict that this increase will be compensated by the more substantial decrease in particle production, we provide new information on the influence of PAP expression in cells that is relevant to understanding its effectiveness as a potential antiviral agent.

## Results and Discussion

### PAP reduces HIV-1 production from cells

We examined the effect of PAP on HIV-1 production by transient transfection of 293 T cells with the proviral clones pMenv(-) or pNL4-3, and 3x-Flag-PAP, 3x-Flag-PAPx or vector control (pcDNA3). PAPx is the enzymatically inactive mutant of PAP that serves as a negative control for PAP activity [19], and immunoblot analysis using a Flag-specific antibody indicated that both PAP and PAPx were expressed in cells (Figure 1A). To assess virus production, media of cells were collected 40 hours following transfection and a p24 CA ELISA was performed. Increasing amounts of PAP plasmid transfected into cells with pMenv(-) reduced the amount of HIV-1 particles in a dose-dependent manner (Figure 1B). p24 CA protein level was extremely low at the highest amount of 3x-Flag-PAP plasmid (1 µg) transfected into cells, such that we used a log scale to illustrate these values. Expression of PAPx did not alter virus production levels relative to vector control (pcDNA3), suggesting that the enzymatic activity of PAP was responsible for inhibition of virus production. The ELISA results were confirmed by immunoblot analysis of virus particles pelleted by ultracentrifugation from equal volumes of media, showing that PAP reduced Gag protein products to undetectable levels (Figure 1C).

**Figure 2:**
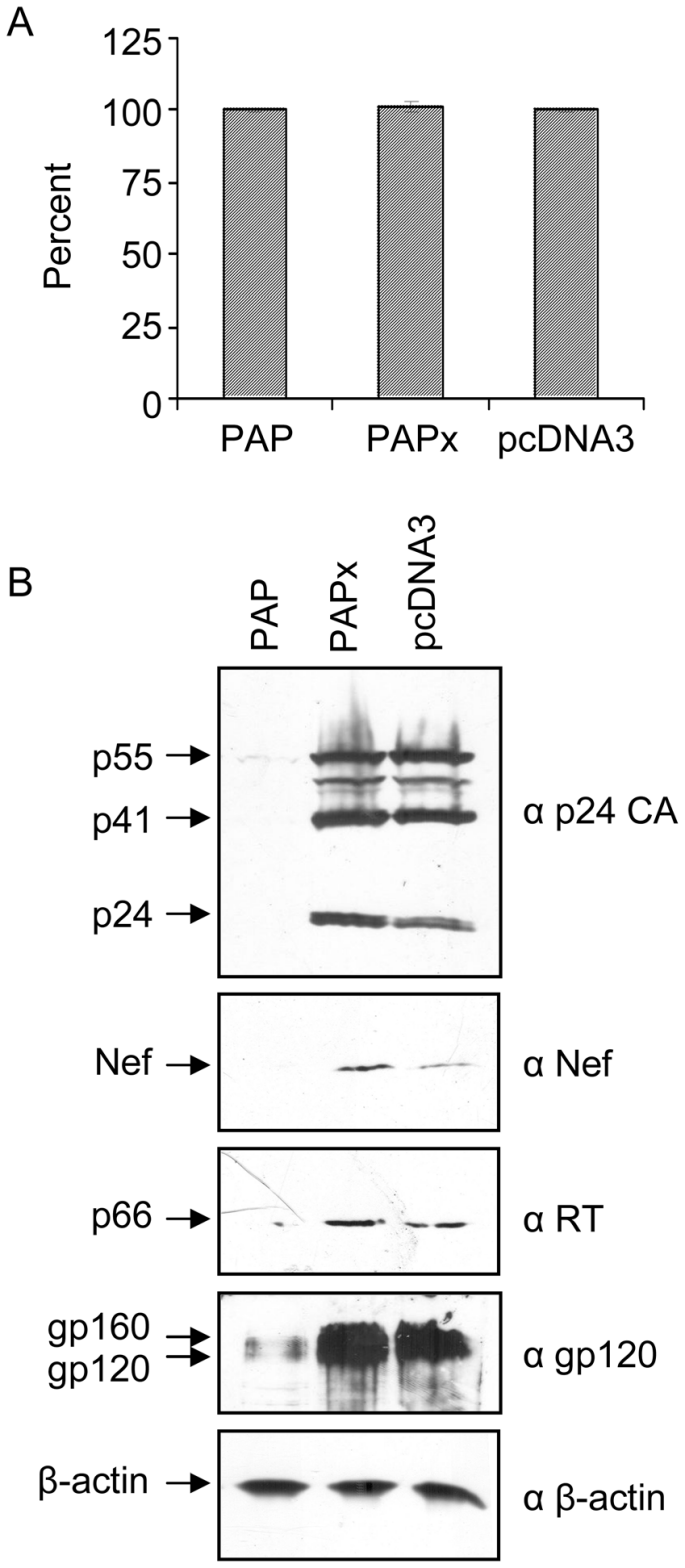
PAP decreases expression of HIV-1 proteins without toxicity to cells. 293 T cells were transfected with the pNL4-3 proviral clone (5 µg) and 2 µg 3x-Flag-PAP, 3x-Flag-PAPx or pcDNA3 vectors. Cells were harvested 40 hours following transfection. (A) Viability was tested by an MTT conversion assay. Values are percentages relative to pcDNA3, as means ± S.E. for three independent experiments. (B) Total cellular protein (150 µg) was separated through 10% SDS-PAGE, followed by immunoblot analysis using p24 CA-specific monoclonal antibody (1∶5,000), or polyclonal antibodies specific for Nef (1∶2,000), RT (1∶2,500) or gp120 (1∶5,000). Samples were probed with β-actin monoclonal antibody (1∶5,000) to control for equal protein loading in each lane. The blots are representative of three separate experiments.

Decrease in virus production was not due to loss of viability of cells expressing PAP. MTT assay results agreed with our previous observations that PAP is not toxic to 293 T cells (Figure 2A; 8). To determine whether reduction in virus production was due to defects in virus assembly or release from cells, the efficiency of virus release was tested by comparing the amount of p24 CA protein in the media to Gag protein synthesized in cells. The amounts of Gag protein products, including p55, p41 and p24, inside cells were assessed by immunoblot (Figure 2B) and ELISA (not shown) using a p24-specific antibody. Consistent with reduction of virus particles released into the media, PAP reduced expression of Gag protein products to barely observable levels inside cells. Therefore, reduction in virus production from cells expressing PAP was likely due to lower expression of Gag protein inside cells, rather than defects in virus assembly or release. The expression of the reverse transcriptase (RT), Nef and Env (gp120) proteins was also decreased in lysates of cells expressing PAP, suggesting that PAP inhibits the expression of both structural and regulatory viral proteins. These data are consistent with a previous study showing that incubation of HIV-1 infected T cells with PAP immunoconjugates reduced the levels of viral proteins in the cells (6); however, the resulting particle characteristics were not assessed.

### PAP increases HIV-1 particle infectivity

**Figure 3:**
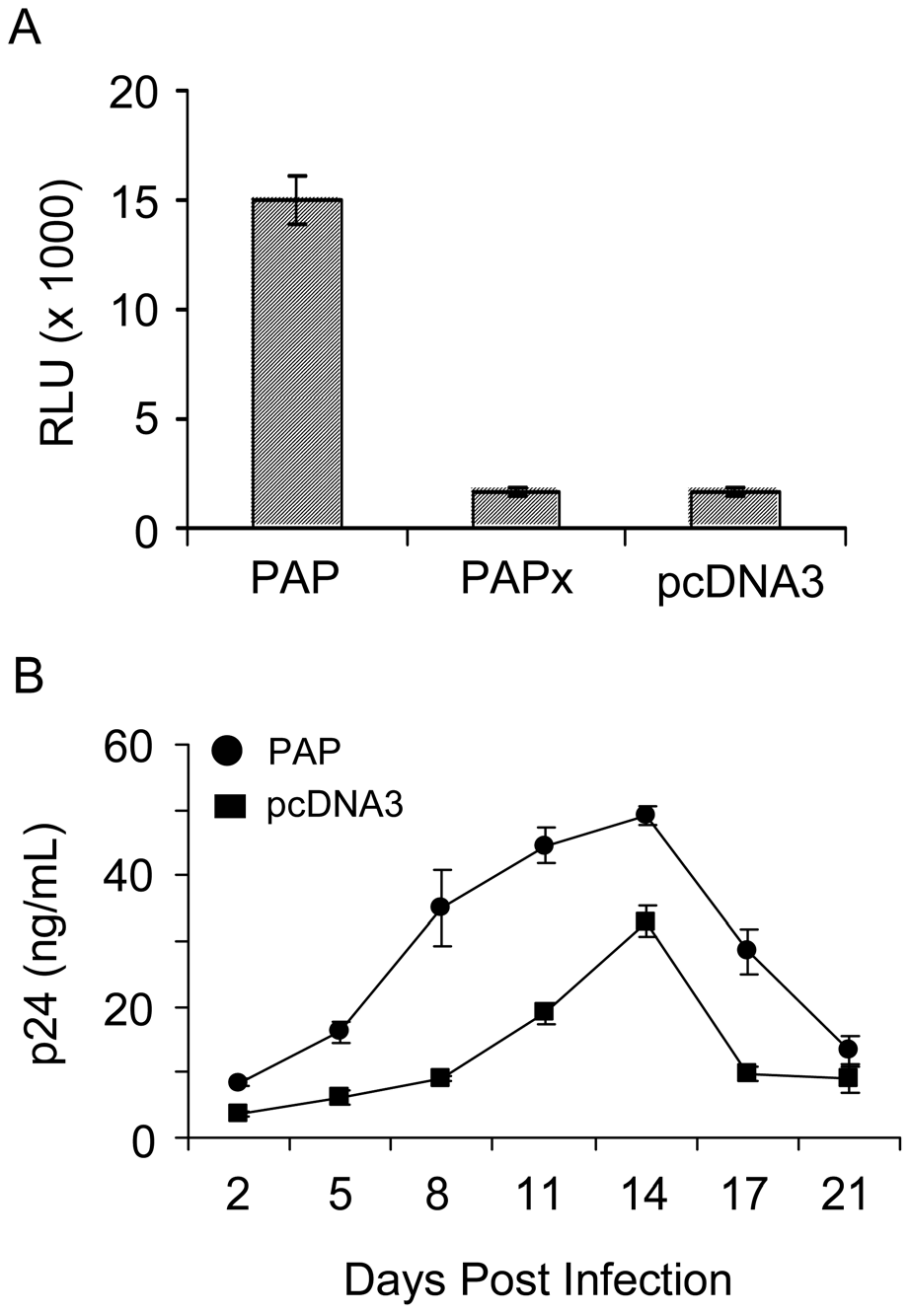
PAP increases HIV-1 particle infectivity. Virus particles were generated by transfection of 293 T cells with the pNL4-3 proviral clone (5 µg) and 0.5 µg 3x-Flag-PAP, 3x-Flag-PAPx or pcDNA3 vectors. (A) Equivalent amounts of purified virus (12 ng) in 1 mL of media were used for a single-cycle infection of 1×10^5^ 1G5 cells in a 24-well plate. The cells were washed 8 hours following infection and treated with AZT (10 µM). Following another 40 hours of incubation, the cells were harvested, lysed and a luciferase assay was performed to determine relative virus infectivity. Values are means ± S.E. from triplicate samples of three different experiments. (B) Equivalent amounts of purified virus (16 ng) in 1 mL of media were used to infect 1×10^5^ MT-2 cells in a 24-well plate. Following 4 hours of incubation, the cells were washed with medium and incubated for another 21 days. Aliquots of media were collected on daily intervals and virus production was determined by p24 CA ELISA. Values are means ± S.E. from triplicate samples of three different experiments. Virus particles used for infection and produced from cells expressing PAP or vector control are indicated by circles or squares, respectively.

As a measure of virus quality, we tested the infectivity of particles released from PAP-expressing cells. Particles were purified from media of 293 T cells co-expressing the proviral clone pNL4-3, and 3x-Flag-PAP, 3x-Flag-PAPx or vector control (pcDNA3). A low amount of PAP (0.5 µg) was transfected into cells to allow sufficient virus production for further analysis. Virus infectivity was assessed by single-cycle infection of the 1G5 reporter cell line with equivalent amounts of particles. The extent of HIV-1 LTR activation was determined by luciferase activity in the lysate of 1G5 cells harvested 48 hours following infection to measure the extent of HIV-1 particle infectivity. Cells were treated with AZT 8 hours following infection to prevent further rounds of infection. Co-expression of PAP and HIV-1 in cells resulted in approximately 7-fold increased infectivity of virus progeny relative to those from cells expressing PAPx or pcDNA3 (Figure 3A).

To confirm the effect of PAP on virus infectivity, we performed productive infection analysis using the human T-lymphoblastoid cell line (MT2). The cells were infected with normalized p24 CA amounts of virus, and replication was monitored by removing aliquots of cell media over a 21-day period and testing for virus production by p24 CA ELISA. Consistent with single-cycle infectivity assays, prior co-expression of PAP with HIV-1 resulted in production of more infectious virus particles (Figure 3B). To determine whether changes in virus infectivity were due to factors released from PAP-expressing cells or a specific feature of virus particles produced from these cells, we also tested the effect of media from cells expressing only PAP on infectivity of purified virus particles produced from cells co-transfected with pcDNA3 and pNL4-3,. This was done by mixing filtered media collected from cells expressing PAP alone with purified virus particles from cells co-transfected with pcDNA3 and pNL4-3. We did not detect any changes in virus infectivity with this treatment (data not shown), suggesting that expression of PAP in virus producing cells may have altered specific components of HIV-1 particles.

### Increased virus infectivity is not due to changes in gag/pol protein levels in HIV-1 particles

**Figure 4:**
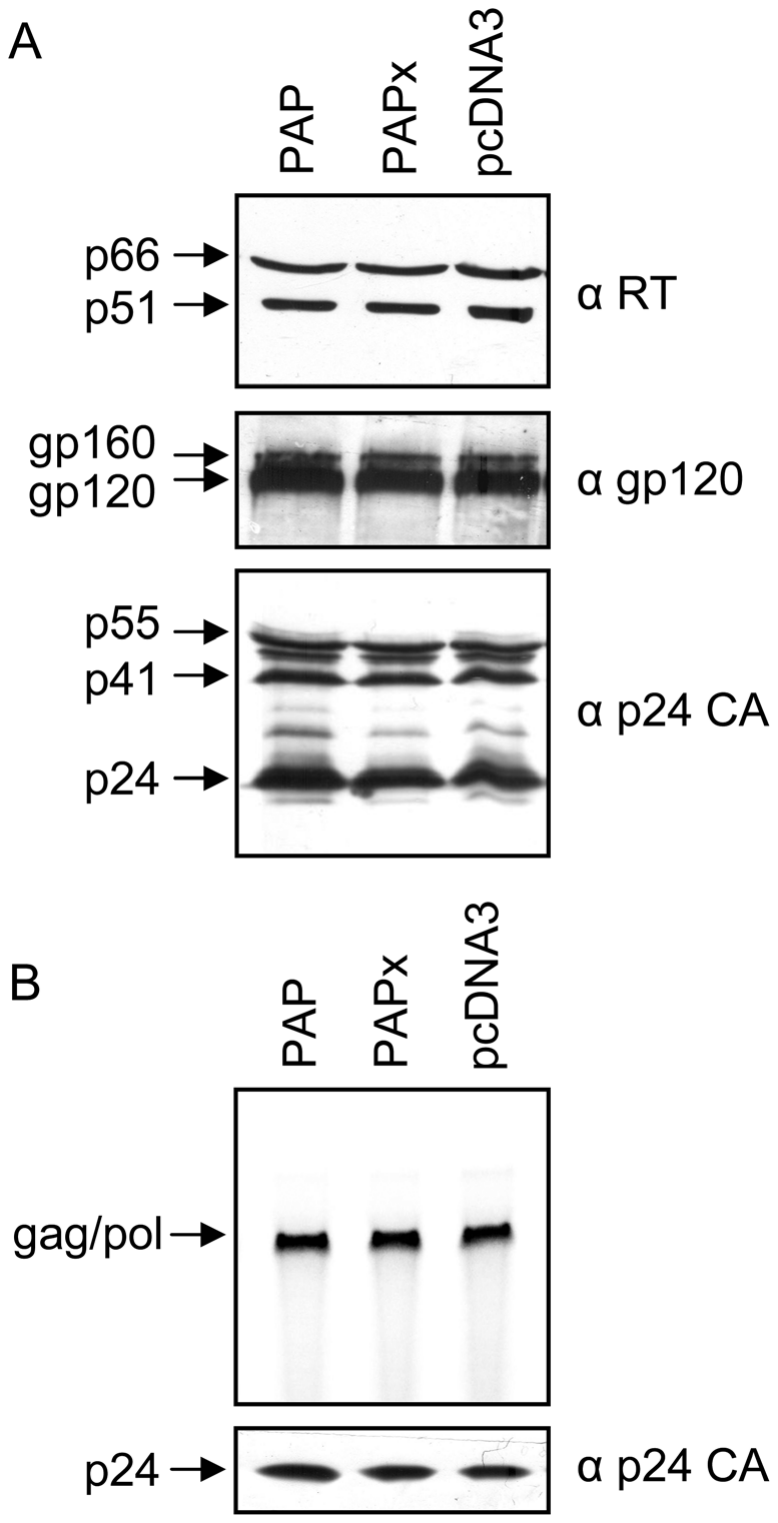
PAP does not alter HIV-1 virus particle composition. Virus particles were collected from media of 293 T cells transfected with the pNL4-3 proviral clone (5 µg) and 0.5 µg 3x-Flag-PAP, 3x-Flag-PAPx or pcDNA3 vectors. (A) Particles were purified by ultracentrifugation through a 20% sucrose cushion and equivalent amounts of purified virus (100 ng) were resolved on 10% SDS-PAGE. Viral proteins were then transferred to nitrocellulose and probed with antibodies specific for RT (1∶2,500), gp120 (1∶5,000) and p24 CA (1∶5,000) proteins. (B) RNA was isolated from equivalent amounts of purified virus particles (100 ng) and analyzed by RNase protection assay using an antisense riboprobe complementary to the gag ORF (nt 600–820). A fraction of the purified viruses was used for immunoblot analysis of p24 CA to control for equal virus pelleting from each sample (lower panel). Blots are representative of five independent experiments performed in triplicate.

To determine the effect of PAP on HIV-1 particle composition, we analyzed the relative amounts of virion-associated reverse transcriptase (RT), envelope glycoprotein (Env) and Gag polyprotein. Proteins from equivalent amounts of highly purified virus particles from media of 293 T cells expressing pNL4-3 and 3x-Flag-PAP, 3x-Flag-PAPx or pcDNA3 were visualized by sequential immunoblot analysis using rabbit anti-RT, sheep anti-gp120 and mouse anti-p24 CA antibodies. No notable differences were detected in the relative amounts of RT, Env and Gag proteins in virions from PAP-expressing cells, suggesting that changes in virus infectivity were not due to alteration in the levels of these proteins in virus particles (Figure 4A). In addition, similar ratios of RT heterodimer (p51*^pol^* and p66*^pol^*), gp120*^env^*, intermediate processing product of Gag (p41*^gag^*) and fully processed Gag (p24 CA*^gag^*) were detectable in virus particles purified from cells expressing PAP, PAPx or pcDNA3 (Figure 4A), suggesting that changes in virus infectivity were not likely due to alteration in the processing of viral proteins and degree of virus maturation.

### HIV-1 RNA packaging efficiency is not affected by PAP

Increased efficiency of genomic (gag/pol) RNA packaging into virus particles has been shown to enhance HIV-1 infectivity [12], since not all virus particles package the full-length RNA. To test whether expression of PAP caused an increase in genomic mRNA packaging into HIV-1 particles, total RNA was isolated from normalized amount of p24 CA virus particles that were purified from media of 293 T cells co-expressing the proviral clone pNL4-3 and 3x-Flag-PAP, 3x-Flag-PAPx or vector control. RNase protection assay on isolated virion RNA indicated no obvious changes in the relative efficiency of viral RNA packaging into virus particles (Figure 4B), suggesting that increased virus infectivity was not likely due to enhanced genomic RNA packaging. To control for equal pelleting of virus particles, immunoblot analysis was performed on a fraction of each sample using a p24 CA-specific antibody (Figure 4B, lower panel).

### PAP increases p17 matrix protein phosphorylation

**Figure 5:**
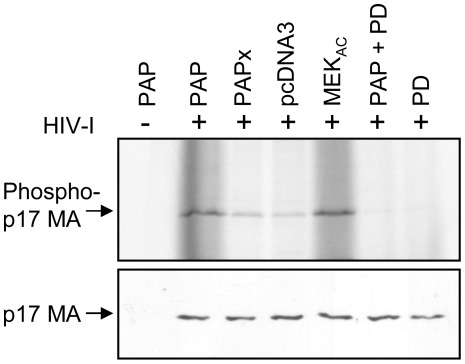
PAP increases the phosphorylation of p17 matrix protein. 293 T cells were transfected with the pMenv(-) proviral clone (5 µg) and 0.5 µg 3x-Flag-PAP, 3x-Flag-PAPx, pcDNA3 or MEK_AC_ vectors. Cells co-transfected with PAP and HIV-1 and cells transfected with HIV-1 alone were additionally treated with 20 µM of the MEK inhibitor PD98059. Particles were purified by ultracentrifugation through a 20% sucrose cushion and equivalent amounts of purified virus (30 ng) were pelleted, lysed and subjected to an endogenous kinase assay using [γ33P-ATP] as a phosphate donor. Samples were resolved on 15% SDS-PAGE and phosphorylated p17 was visualized using a phosphorimager. Fractions of pelleted virus were analyzed via immunoblot using a p17-MA-specific antibody (1∶1,000). Blots are representative of four separate experiments.

A number of viruses, including HIV-1, incorporate cellular signaling molecules, referred to as virus-associated protein kinases (VAPKs), within their particles [20]. VAPKs, such as ERK2 MAPK, have been shown to play a role in HIV-1 infectivity by phosphorylating p17 MA protein [15], which facilitates the nuclear import of HIV-1 preintegration complexes [17]. Since the main substrate for ERK2 within HIV-1 particles is the p17 MA protein, the effect of PAP expression on the activation of ERK2 was assessed by co-transfecting the proviral clone pMenv(-) with 3x-Flag-PAP, 3x-Flag-PAPx or vector control and testing the extent of MA phosphorylation inside virus particles by an *in vitro* kinase assay. Equivalent amounts of purified virus from media were used for this kinase assay, and as shown in Figure 5, increase of a phospho-protein was visible in HIV-1 particles produced from PAP-expressing cells, but not from cells transfected with PAPx or vector control. As an experimental control, supernatant of PAP-expressing cells without the virus was also ultracentrifuged and tested in the kinase assay. However, no radiolabeled proteins were detected in this sample, suggesting that the observed band was virus-specific, corresponding to the molecular weight of phosphorylated MA protein.

To determine whether phosphorylation of putative p17 MA inside virus particles produced from cells expressing PAP was likely due to higher levels of active ERK2 inside particles, the extent of virion protein phosphorylation was analyzed by treatment of cells co-expressing the proviral clone pMenv(-) and 3x-Flag-PAP with PD98059 [2-(2′-amino-3′-methoxyphenyl)oxanaphthalen-4-one]. PD98059 is a specific inhibitor of MAPK activating enzyme, MAPK kinase (MEK) [21], which prevents activation of MAPK and subsequent phosphorylation of MAPK substrates [22]. Incorporation of γ^33^P-ATP into virion-associated p17 MA was reduced upon treatment of cells with PD98059, suggesting that increased p17 MA phosphorylation was due to higher levels of active ERK2 inside purified HIV-1 particles produced from cells expressing PAP (Figure 5, upper panel). Consistent with previous reports, PD98059 did not significantly lower virus production from cells as tested by ELISA (data not shown). As an experimental control, viruses were purified from media of 293 T cells co-transfected with HIV-1 proviral clone and a plasmid encoding constitutively active MEK (MEK_AC_). As expected, viruses produced from these cells showed a higher degree of p17 MA phosphorylation compared to cells co-transfected with pcDNA3 (Figure 5, upper panel). A portion of the pelleted virions was used for immunoblot analysis using anti-p17 MA antibody to ensure similar amounts of virus particles in the kinase assay samples (Figure 5, lower panel). These results suggest that expression of PAP inside cells increased the activity of ERK2 within HIV-1 particles. Our attempts at immunoblot analysis of phospho-ERK2 inside virus particles were unsuccessful, because the amount of virus produced from PAP-expressing cells was too low to allow detection.

### PAP increases the level of active MEK and ERK1/2 inside cells

**Figure 6:**
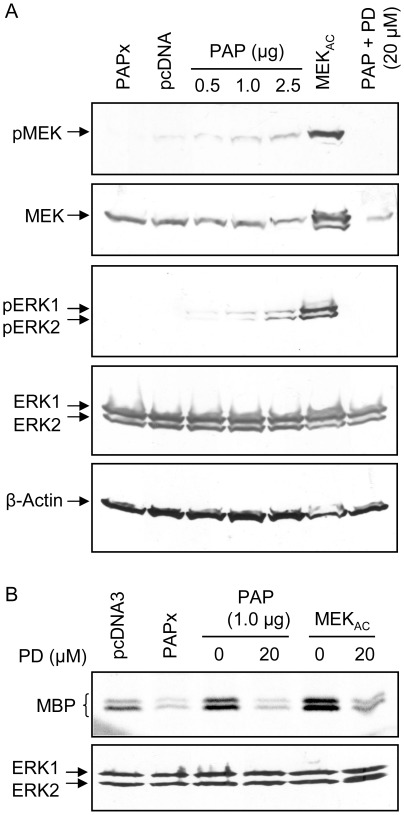
PAP activates ERK1/2 MAPK in cells. (A) 293 T cells were transfected with 3x-Flag-PAP (0.5, 1.0, or 2.5 µg), 3x-Flag-PAPx (2.5 µg), pcDNA3 (2.5 µg) or MEK_AC_ (1.0 µg) vectors. 24 hours prior to transfection, cells were refed with DMEM without FBS until they were harvested. A portion of cells transfected with 3x-Flag-PAP (2.5 µg) was treated with the MEK inhibitor PD98059 (20 µM). Total cellular protein (100 µg) was resolved through 12% SDS-PAGE, followed by sequential immunoblot analysis using antibodies specific for phospho-MEK (1∶2,000), MEK (1∶2,000), phospho-ERK1/2 (1∶2,000), ERK1/2 (1∶2,000) and β-actin (1∶5,000). (B) Total ERK1/2 was immunoprecipitated from lysate of cells (300 µg total protein), followed by a kinase assay using MBP as substrate. Cells transfected with 3x-Flag-PAP (1.0 µg) or MEK_AC_ (1.0 µg) were also treated with 20 µM PD98059 MEK inhibitor. Phosphorylated proteins were visualized with a phosphorimager. A fraction of immunoprecipitated proteins was analyzed by immunoblotting using an ERK1/2 specific antibody (1∶5,000). Blots are representative of four independent experiments.

Activation of the cellular ERK1/2 MAPK signaling pathway by extracellular stimuli has been shown to increase the level of phosphorylated ERK2 packaged inside HIV-1 particles [15], which in turn, enhanced virus infectivity. To determine whether PAP activated MEK and ERK1/2 in cells, we transfected 293 T cells with 3x-Flag-PAP and compared the level of phosphorylated MEK and ERK1/2 to cells transfected with 3x-Flag-PAPx, pcDNA3 or MEK_AC_ (encoding constitutively active MEK). Expression of PAP increased the level of phospho-MEK by a small degree relative to cells transfected with PAPx or pcDNA3 (Figure 6A). As expected, activation of MEK was substantially less than that observed for cells expressing the constitutively active form of the protein (MEK_AC_). The level of total MEK remained unchanged in PAP-expressing cells, indicating that PAP enhanced the activity of MEK, rather than increasing MEK synthesis. To determine whether this minor degree of MEK activation affected its downstream target ERK1/2, we also tested activation of ERK1/2 in the presence of PAP. Expression of PAP increased the level of active phosphorylated ERK1/2 in a concentration dependent manner, while the total amount of ERK1/2 inside the cells remained unaffected (Figure 6A). The double bands seen in all the lanes represented the two isoforms ERK1 (44 kDa) and ERK2 (42 kDa). Maximum ERK1/2 phosphorylation was detected in the positive control lane (MEK_AC_). Although the increase in ERK1/2 phosphorylation was moderate relative to constitutively active MEK, it appeared consistently in several replicate assays and was effectively reduced by the MEK specific inhibitor PD98059.

To confirm that ERK1/2 was more active in cells expressing PAP, an *in vitro* kinase assay was performed on lysates of 293 T cells expressing 3x-Flag-PAP, 3x-Flag-PAPx, pcDNA3 or MEK_AC_. Total cellular ERK1/2 was immunoprecipitated from cell lysates using antibodies specific for ERK1/2, followed by addition of myelin basic protein (MBP) as substrate for ERK1/2 and γ ^33^P-ATP as phosphate donor. As indicated in Figure 6B (upper panel), lysate of cells expressing PAP showed a higher degree of MBP phosphorylation than cells expressing PAPx or pcDNA3, although similar amounts of ERK1/2 precipitated from each sample (Figure 6B, lower panel). Greater phosphorylation of MBP was achieved in immunoprecipitated lysate of cells expressing constitutive MEK (MEK_AC_), which was consistent with the result from immunoblot analysis of phospho-ERK1/2 levels. Furthermore, treatment with PD98059 of cells transfected with MEK_AC_ or 3x-Flag-PAP reduced the level of MBP phosphorylation, suggesting that phosphorylation was due to active ERK1/2 immunoprecipitated from lysates of cells (Figure 6B). The immunoblot analysis and kinase assay indicated that PAP activated ERK1/2 MAPK, and activation of this kinase may be responsible for increased virus infectivity.

It is uncertain how PAP may be activating ERK1/2, but previous studies have shown that several ribosome inactivating proteins cause the phosphorylation of kinases involved in the MAPK pathway, most often resulting in cell death [23]–[25]. Cytotoxicity, in turn, has been used to explain antiviral activity, as host cell death would diminish virus proliferation. For example, the antiviral activity of trichosanthin, a ribosome inactivating protein, is attributed to its ability to reduce levels of the anti-apoptotic protein Bcl-2 [26], thought to be controlled at the transcriptional level by members of the MAPK pathway upstream of cAMP response element binding protein (CREB) [27]. These findings agree with earlier work showing that trichosanthin induces apoptosis in HIV-1 infected cells [28]. In contrast with most ribosome inactivating proteins, PAP expression in cultured cells does not cause cellular changes characteristic of programmed cell death, such as cleavage of caspase-3 and PARP and increased cell permeability [29]. We have shown previously that PAP activates the JNK/SAPK pathway but not p38/HOG MAPK signaling pathways in 293 T cells [29]. The activation is triggered by depurination of the 28S rRNA, which mediates a signal that is transduced by the JNK/SAPK pathway [30]. However, PAP does not decrease cell viability or cellular translation rate, likely because a small percentage (16%) of ribosomal RNA is depurinated [8].

### Activation of ERK2 by PAP contributes to increased virus infectivity

**Figure 7:**
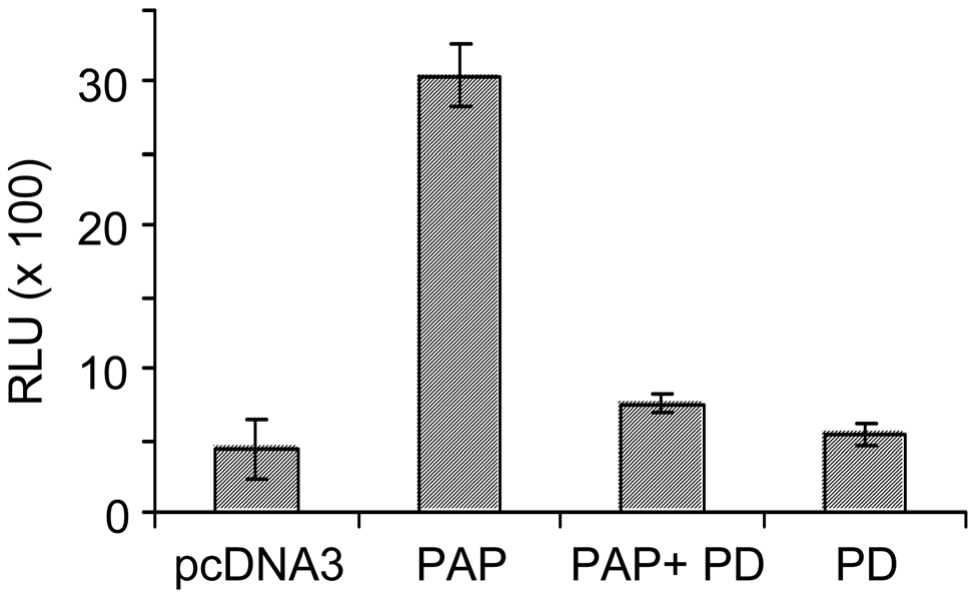
Infectivity of HIV-1 particles decreases upon treatment with MEK inhibitor. 293 T cells were transfected with 5 µg pNL4-3 and 0.5 µg 3x-Flag-PAP or pcDNA3 plasmids. Cells transfected with 3x-Flag-PAP plasmid or untransfected were also treated with PD98059 (20 µM). Virus particles were purified from the media of cells collected 40 hours following transfection and equivalent amounts of virus particles (3 ng) were used to infect 1×10^5^ 1G5 cells. Cells were washed and re-fed with fresh medium containing AZT (10 µM) 8 hours following infection and incubated for an additional 40 hours. Cells were harvested, lysed and a luciferase assay was performed to determine relative virus infectivity. Values are means ± S.E. from triplicate samples of three different experiments.

To test that modulation of ERK2 activity inside virus particles produced from cells expressing PAP altered virus infectivity, cells co-expressing PAP and HIV-1 (pNL4-3) were treated with MEK inhibitor (PD98059) 18 hours following transfection. Equivalent amounts of purified virus particles collected from these cells were then used to infect the 1G5 reporter cell line. Cells were treated with AZT 8 hours following infection to ensure that only a single round of infection occurred while cells were incubated for an additional 40 hours. Analysis of luciferase activity in lysates of these cells indicated that while expression of PAP caused an increase in virus infectivity, treatment of PAP-expressing cells with PD98059 reduced virus infectivity to levels similar to virus particles produced from cells transfected with pcDNA3 (Figure 7). As expected, PD treatment of cells not expressing PAP did not alter infectivity of released virions. These data suggest that activation of ERK2 contributed to increased infectivity of virus particles generated from PAP-expressing cells.

### PAP reduces productive HIV-1 infection

**Figure 8:**
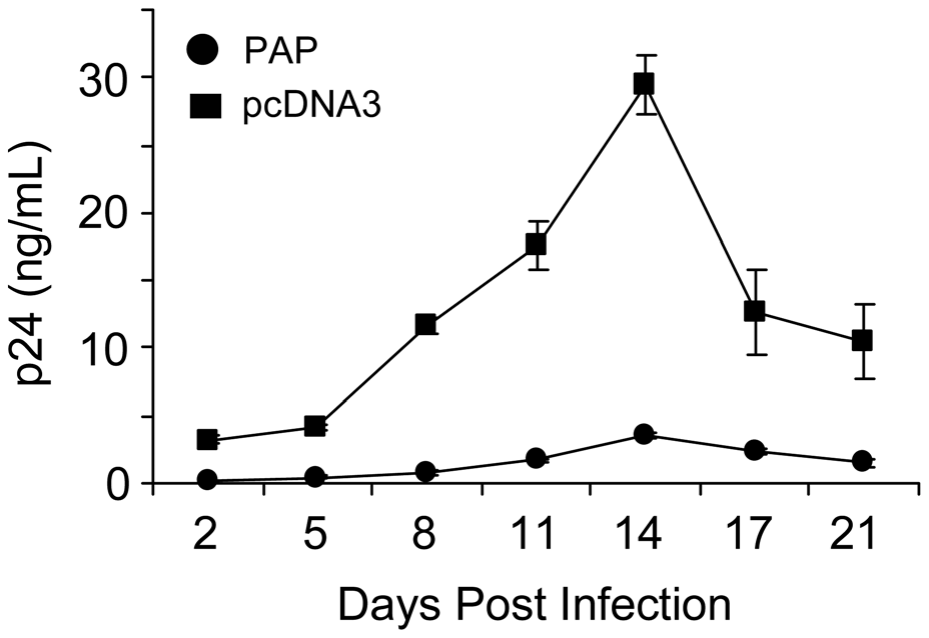
Decreased synthesis of HIV-1 particles by PAP reduces productive infection. Equivalent volume of medium (200 µL) containing virus from 293 T cells transfected with the pNL4-3 proviral clone (5 µg) and 0.5 µg 3x-Flag-PAP or pcDNA3 vectors was used to infect 1×10^5^ MT-2 cells in a 24-well plate. Following 4 hours of incubation, the cells were washed with fresh medium and incubated for another 21 days. Aliquots of media were collected every three days and virus production was determined by p24 CA ELISA. Medium from cells expressing PAP or vector control is indicated by circles or squares, respectively. Values are means ± S.E. from triplicate samples of three different experiments.

Results from our study show that although co-expression of PAP with HIV-1 substantially lowered the production of virus, these particles were more infectious, likely due to increased ERK1/2 MAPK activation. We then asked whether the combined effect of PAP on virus production and infectivity would result in greater virus production upon *de novo* infection of cells over time. For this assay, equal volumes of media from cells expressing pNL4-3 with PAP or pcDNA3 were used to infect MT-2 cells and virus production from these cells was assessed over a 21-day period using a p24 CA ELISA (Figure 8). MT2 cells infected with medium from PAP-expressing cells produced significantly less virus over this time compared with medium from vector control cells. Therefore, although PAP enhanced virus infectivity, this effect was compensated by the low amount of virus released from PAP-expressing cells.

When evaluating the potential of PAP as an antiviral agent, it is important to assess its effects on the host cell in addition to the virus. We acknowledge that our experiments were conducted entirely in cell lines and that they would need to be confirmed in primary cells; however, the transfection of 0.5 µg of PAP DNA into 293 T cells caused a 7-fold increase in particle infectivity, as measured by a single-cycle infection. This same amount of transfected PAP DNA increased phosphorylation levels of MEK and ERK1/2 to a minor extent above those of negative control cells, indicating that even modest modulation of cellular proteins can have substantial effect on stages of the virus life cycle. In addition to increasing virus infectivity, PAP significantly decreased particle production. Namely, transfection of the same amount of PAP DNA, which represented a level of protein just visible by immunoblotting, reduced virus particle production by approximately 450-fold. Therefore, increased infectivity may not contribute significantly to the progress of infection if virus levels are barely detectable by ELISA. Even though PAP is not expressed to a high degree in 293 T cells, it substantially suppresses HIV-1 production, suggesting that the protein has potential as an effective inhibitor of HIV-1 replication at low concentrations.

## Materials and Methods

### Cell lines

293 T cells (ATCC, catalog # CRL-11268) were maintained in Dulbecco's Modified Eagle's Medium (DMEM) supplemented with 10% heat-inactivated fetal bovine serum (FBS), penicillin (100 U/mL) and streptomycin (100 µg/mL). 293 T cells used to determine MEK and ERK1/2 levels and activity were maintained in the same manner as described above until 24 hours prior to transfection. At this time, the cells were grown in DMEM without FBS until they were harvested. The HIV-1 indicator cell line 1G5 (NIH AIDS Reference and Reagent Program, catalog # 1819) is a derivative of Jurkat cells stably transfected with a reporter construct containing the HIV-1 long terminal repeat (LTR) upstream of the luciferase gene [31]. These cells were maintained in RPMI 1640 medium, supplemented with 10% FBS, penicillin (100 U/mL) and streptomycin (100 µg/mL). Human lymphoblastoid T-cell line MT-2 (NIH AIDS Reference and Reagent Program, catalog # 237) was maintained in RPMI 1640, supplemented with 10% FBS, penicillin (100 U/mL) and streptomycin (100 µg/mL). All cells were cultured at 37°C with 5% CO_2_.

### Plasmid constructs and antibodies

Plasmids 3x-Flag-PAP and 3x-Flag-PAPx were described previously [29]. The proviral clones pMenv(-) (catalog # 2089) and pNL4-3 (catalog # 114) were obtained from NIH AIDS Reference and Reagent Program. The plasmid pMenv(-) has been described previously [32] and results in production of non-infectious HIV-1 particles. The plasmid encoding constitutively active MEK (MEK_AC_; ΔN3 S218D/S222E; [33]) was generously provided by Dr. J. C. McDermott (York University, Canada).

Polyclonal antibodies specific for MEK (catalog # 9122), phospho-MEK (catalog # 9154), ERK1/2 (catalog # 9102) and phospho-ERK1/2 (catalog # 4377) were purchased from Cell Signaling. Mouse anti-p24 CA (catalog # 6457), rabbit anti-RT (catalog # 6195), anti-Nef (catalog # 2949), anti-p17 MA (catalog# 4811) and sheep anti-gp120 (catalog # 288) were obtained from NIH AIDS Reference and Reagent Program. Mouse M2 anti-Flag antibody (catalog # F3165) was purchased from Sigma Aldrich.

### Transfection, MTT assay, and generation of virus stocks

293 T cells were transiently transfected using the calcium chloride transfection method, as described previously [29]. Briefly, 5×10^6^ cells in 10-cm plates were transfected with 5 µg proviral DNA (pNL4-3 or pMenv(-)) and the indicated amounts of 3x-Flag-PAP, 3x-Flag-PAPx, pcDNA3 or pMEK_AC_. Cellular viability was judged using an MTT conversion assay (Roche Applied Science) as per the manufacturer's instructions. Approximately 40 hours after transfection, cells were seeded into a 96- well plate and MTT reagent (20 µL) was added to each well. The plate was incubated at 37°C for 4 hours after which 100 µL of Solubilizing buffer was added to each well and the plate was incubated at 37°C for an additional for 4 hours. Following incubation, absorbance was measured at 595 nm.

The media of cells were collected or cells were harvested 48 hours following transfection for virus purification or protein expression analysis, respectively. Media collected from cells were clarified by centrifugation at 3,000× g for 10 minutes, filtered through a 0.22 µM filter, and then pelleted through a 20% sucrose cushion by ultracentrifugation at 184,000× g for 90 minutes at 4°C. Pelleted virus particles were resuspended in buffers appropriate for subsequent experiments or aliquoted and then stored at −80°C for use in subsequent experiments. To generate virus particles from cells treated with PD98059 (catalog # 9900, Cell Signaling), the media of cells were replaced 18 hours following transfection with fresh media containing PD98059 (20 µM), and media were collected 24 hours later for virus purification and quantification. The amount of virus in each sample was determined by p24 CA ELISA (catalog # BP205, NCI, Frederick, MA) following manufacturer's instructions.

### Infectivity assays

Single-cycle infectivity assays were performed using 1G5 reporter cells that stably express the luciferase gene downstream of HIV-1 LTR. Cells were seeded at a density of 1×10^5^ cells per well in a 24-well plate immediately prior to infection with HIV-1 virions (12 ng) normalized according to p24 CA amounts. Infections were performed in the presence of 8 µg/mL polybrene in five replicates. The cells were washed 3 times with 1× PBS 8 hours following infection and re-suspended in fresh complete media containing AZT (10 µM) and incubated for an additional 40 hours at 37°C. The cells were harvested and luciferase activity in cell lysates was measured using a luciferase assay system (catalog # E4030, Promega) following manufacturer's recommendations. MT-2 (1×10^5^ cells) were infected in 24 well plates with equivalent amounts of virus (16 ng) or equal volume of media (200 µL) containing different amounts of virus, in the presence of 8 µg/mL polybrene. Following a 4-hour incubation, the cells were washed 3 times with 1× PBS and cultured in complete media for a period of 21 days. Aliquots of cell supernatants were removed every 3 days and the amount of virus produced was estimated with p24 CA ELISA.

### Immunoblot analysis

Cells were harvested and lysed in lysis buffer (25 mM HEPES-KOH pH 7.4, 2 mM EGTA, 1 mM DTT, 10% glycerol, 1% Nonidet P-40) for 10 minutes on ice. Cells used to show PAP expression were lysed using nuclear lysis buffer (25 mM HEPES-KOH pH 7.4, 2 mM EGTA, 1 mM DTT, 10% glycerol, 1% Nonidet P-40, 1% SDS). Both lysates were thoroughly vortexed and clarified by centrifugation at 14,000× g for 10 minutes at 4°C to remove cell debris and proteins were visualized by immunoblot analysis as described previously [34]. Briefly, 70–150 µg of cell lysate protein was resolved on a 10–12% SDS-PAGE, transferred to nitrocellulose and then probed with the indicated antibodies. Immunoblot analysis of virus particles was performed by dissolving pelleted virions in 2× SDS sample buffer, followed by separation of viral proteins on a 10–12% SDS-PAGE and probing with the indicated antibodies.

### Virus protein phosphorylation analysis

Equivalent amounts of purified virus particles were pelleted by centrifugation at 16,000× g at 4°C for 2 hours. Virus pellets were then resuspended in 20 µL of reaction buffer (20 µCi of [γ-^33^P]ATP, 50 mM HEPES-KOH pH 7.4, 10 mM MgCl_2_, 0.5% Nonidet P-40), followed by incubation at 30°C for 30 minutes. The reaction was stopped by adding equal volume of 2× SDS sample buffer and heating at 90°C for 5 minutes. Samples were then resolved through 12% SDS-PAGE and visualized using a phosphorimager (BioRad). Equal fractions of the pelleted virus particles were dissolved in 2× SDS sample buffer and resolved through 12% SDS-PAGE, followed by immunoblot analysis using anti-p24 CA antibody (1∶5,000).

### In vitro kinase assays

Cells were harvested 40 hours following transfection and lysed in lysis buffer (25 mM HEPES-KOH pH 7.4, 2 mM EGTA, 1 mM DTT, 10% glycerol, 1% Nonidet P-40, 1% phosphatase inhibitor cocktail [Sigma], 1× complete protease inhibitor cocktail [Roche]). The lysates were cleared by centrifugation at 16,000× g for 10 minutes at 4°C, following which 300 µg of total cellular protein was immunoprecipitated with anti-ERK1/2 antibody (1∶1,000) for 4 hours at 4°C with constant agitation. Protein A Sepharose beads (20 µL) were then added and samples were agitated for another hour at 4°C, followed by a series of washes; 25 mM HEPES–KOH pH 7.4 (buffer 1); buffer 1 containing 0.25 M NaCl (buffer 2); buffer 1 containing 0.25 M NaCl and 1% Triton X-100; then buffer 2 again followed by buffer 1; culminating with two washes in kinase buffer (20 mM MOPS pH 7.2, 10 mM MgCl_2_, 2 mM EGTA, 1 mM DTT, 0.1% Nonidet P-40). An aliquot of the beads was removed and solubilized in 2× SDS sample buffer, followed by separation through 12% SDS-PAGE and immunoblot analysis using anti-ERK1/2 antibody (1∶5,000). The remainder of the beads was used in a kinase assay to measure ERK1/2 activity in cell lysates. Reactions were performed in a total volume of 20 µL kinase buffer containing 1.5 mM rATP, 2 µg myelin basic protein (MBP) as substrate and 10 µCi/mL [γ-^33^P]ATP. The samples were incubated at 30°C for 30 minutes and the reaction was stopped by adding equal volume (20 µL) of 2× SDS sample buffer. Samples were separated through 12% SDS-PAGE and phosphorylated proteins were visualized with a phosphorimager (BioRad).

### RNase protection assay

Purified virus particles (equivalent to 150 ng of p24 CA) were pelleted by centrifugation at 16,000× g for 2 hours at 4°C and total RNA was isolated using TriReagent (Bioshop) following manufacturer's recommendations. RNA was precipitated with 10 µg tRNA added to each sample as a carrier. The RNase protection assay was performed exactly as described previously [8], using a radiolabeled antisense riboprobe complementary to the gag open reading frame (ORF) of HIV-1. The probe template was generated by PCR amplification of nucleotides 600 to 820 of the HXB2 proviral clone (Accession # K03455.1) using HXB2-600F: 5′-CCCCAAGCTTAGACCCTTTTAGTCAGTGTGGAAAATCTCTAGC-3′ forward and HXB2-820R: 5′-CCCCGAATTCTAATACGACTCACTATAGGCCCC TTAATACTG -3′ reverse primers. The reverse primer also contained the T7 RNA polymerase promoter sequence, which was used to transcribe the negative-strand RNA probe in the presence of 50 µCi of [α-^33^P]CTP.
